# Increased fat mass index is associated with decreased glomerular filtration rate estimated from cystatin C. Data from Malmö Diet and Cancer cohort

**DOI:** 10.1371/journal.pone.0271638

**Published:** 2022-07-21

**Authors:** Agne Laucyte-Cibulskiene, Peter M. Nilsson, Gunnar Engström, Anders Christensson

**Affiliations:** 1 Department of Clinical Sciences, Lund University, Skane University Hospital, Malmö, Sweden; 2 Department of Nephrology, Lund University, Skane University Hospital, Malmö, Sweden; 3 Cardiovascular Epidemiology, Department of Clinical Sciences, Lund University, Malmö, Sweden; Istituto Di Ricerche Farmacologiche Mario Negri, ITALY

## Abstract

**Background:**

This study aims to describe associations of obesity and CKD in a Swedish urban population. The impact of fat mass, from bioimpedance analysis, on eGFR based on cystatin C and/or creatinine is studied.

**Methods:**

5049 participants from Malmö Diet and Cancer Study the cardiovascular arm (MDCS‐CV) with available body mass composition (single frequency bioimpedance analysis) and cystatin C measured at baseline were selected. Body mass index (kg/m^2^) was used to define overweight/obesity. eGFR was calculated using cystatin C (eGFR_CYS_) and creatinine (eGFR_CR_) equations: Chronic Kidney Disease Epidemiology Collaboration 2012 (CKD-EPI_CR,_ CKD-EPI_CYS_, CKD-EPI_CR-CYS_)_,_ eGFR_CYS_ based on Caucasian, Asian, pediatric, and adult cohorts (CAPA), the Lund-Malmö revised equation (LMrev), and Modified Full Age Spectrum creatinine-based equation (EKFC_CR_). Two different fat mass index (FMI) z-scores were calculated: FMI z-score_Larsson_ and FMI z-score_Lee_.

**Results:**

Lower eGFR_CYS_ and eGFR_CR-CYS_ following multiple adjustments were prevalent in overweight/obese subjects. Increase in FMI z-score_Larsson_ or FMI z-score_Lee_ was related to decrease in predicted CAPA, CKD-EPI_CYS_, CKD-EPI_CR-CYS_ and CAPA-LMrev equation.

**Conclusion:**

eGFR_CYS_, in contrast to combined eGFR_CR-CYS_ and eGFR_CR_, demonstrate the strongest association between FMI and kidney function.

## Introduction

The growing burden of obesity worldwide and its associations with chronic kidney disease (CKD) is becoming a hot topic for the renal medicine community. Actually, obesity and CKD share the same mechanisms such as renin-aldosterone-angiotensin system alteration [1], hyperinsulinemia, inflammation and oxidative stress [2–4]. The obesity related inflammation [5] enhances athero-/arteriosclerosis and kidney damage3, and on the opposite, CKD leads to an inflammatory state [6] causing cardiovascular dysfunction.

CKD diagnosis using creatinine-based estimated glomerular filtration rate (eGFR) tends to be inaccurate in obese subjects [7]. Cystatin C (≅13.3 kDa), a cysteine protease inhibitor, has been proposed as an alternative biomarker for evaluating kidney function and unlike creatinine is not dependent on muscle mass [8]. Cystatin C is also a stronger predictor of cardiovascular diseases compared to creatinine [9]. An increase of cystatin C in cardiovascular outcomes might be compensatory to increased cathepsin S (19–23 kDa) activity in atherosclerotic plaques [10]. Besides, cathepsin S was discovered in obese subjects [11] pointing out the potential shared pathophysiologic pathways between obesity and cardiovascular disease [12], and/or identifying altered glomerular permeability and consequently leading to serum cystatin C accumulation.

Cystatin C expression in human adipose tissue could in part explain increased serum cystatin C levels in obesity [13, 14]. Besides, fat accumulation per se [15, 16], determined by bioelectrical impedance analysis, has been shown to be associated with CKD estimated by creatinine based eGFR equations and needs further clarification. Based on the current knowledge, we *aimed* to identify relationship between fat mass and kidney function measured by different cystatin C and creatinine eGFR equations, and to determine certain sex-specific link between body composition and kidney function.

Hereby, we hypothesize that tools for fat mass evaluation acquired by bioimpedance analysis are strongly associated with eGFR based on cystatin C and indicate sex-specific relationship in a Swedish urban population.

## Materials and methods

### Study design and settings

Subjects who participated in the population-based Malmö Diet and Cancer Study (MDCS) [17] were selected to this observational cohort study. MDCS included 17 035 women and 11 063 men, born 1923–1950 and residing in Malmö. MDCS was initially designed as a prospective case-control study that aimed to explore influence of western diet on the incidence of certain forms of cancer after follow-up.

### Participants

During the year 1991–1996 MDCS participants were randomly invited to participate in the cardiovascular arm (MDCS‐CV; n = 6103), see Hedblad B *et al*. [17]. We selected those participants who had their body mass composition, creatinine, and cystatin C (n = 5049) measured at the baseline. None of them had cancer at the inclusion to the study.

For more detailed participant recruitment and data collection see Lahmann P *et al*. [18].

### Measurements

#### Body composition and anthropometric measurements

The anthropometric and body composition measurements were performed in a non-fasting state. Weight (kg), height (m), waist circumference (WC) and hip circumference (HC) in cm were measured by a trained nurse as previously described by Lahmann P *et al*. [19]. Body mass index (BMI) calculated as weight in kilograms divided by height in meters squared (kg/m^2^). BMI <25 kg/m^2^ determined as normal, 25 ≤BMI<30 kg/m^2^ as overweight, and >30 kg/m^2^ as obesity. Waist to hip ratio calculated as WC in cm divided by HC in cm (cm/cm).

Diabetes was defined as diabetes mellitus type 1 or type 2 or fasting plasma glucose concentration greater or equal 7 mmol/L. Hypertension was considered as the presence of diagnosis or the use of antihypertensive medication.

Body composition analysis was performed by using single frequency (50 kHz) bioimpedance equipment, BIA-103 RJL system analyzer (RJL Systems, Detroit, MI), according to procedures provided by the manufacturer (tetrapolar electrode placement, subjects in a supine position). Fat mass (FM) (kg), body fat percent (%), lean mass (LM) (kg), total body water (TBW; liter, L) were estimated automatically by using algorithms implemented in BIA-103 RJL system analyzer. Fat mass index (FMI) calculated as FM in kilograms divided height in meters squared (kg/m^2^).

Systolic and diastolic blood pressure (SBP and DBP, mmHg) measured by trained nurses during 1991 to 1996. Mean arterial pressure (MAP) was calculated as follows: SBP+2DBP/3 (mmHg).

### Fat mass index z-score calculation

Recently Lee MM et al. [20] published sex specific reference values for FMI and skeletal muscle mass index (SMMI) in the white ethnic population older than 40 years. They used single-frequency bioimpedance equipment (Tanita BC-418 MA) and developed reference values based on measurements in 390 565 UK adults. Meanwhile, Larsson I et al. [21] provided reference values on body composition (FM, FMI, body fat percent) based on dual-energy X-ray absorptiometry (DEXA) measurements in adult Swedes. They pooled data from four population-based studies: The young adult, The Mölndal Metabolic study, The Swedish Obese Subjects reference study and The Geriatric and Gerontologic Population Study and the Population Study of Women). In total 623 men and 801 women that underwent the anthropometric and DEXA measurements in between 1990 and 2006 were analyzed.

Based on these reference values we calculated two different FMI z-scores. One z-score FMI_Lee_ by using FMI reference values for bioimpedance [20] and the other z-score FMI_Larsson_−by using DEXA provided references [21].

The equation for z-score calculation was as follows:

$$\text{z-score}\left(\text{x}\right)=\left(\text{x}-\mu \right)/\sigma$$

Where x is a raw value, μ –is the population mean (reference value), and σ –is the population standard deviation. For example z-score FMI_Larsson_ = (FMI–FMI_Larsson_)/ σ_Larsson_.

### Biochemistry

Participants donated fasting blood samples that were drawn and immediately frozen to −80°C and stored in a biobank as reported previously [22]. Plasma creatinine (μmol/L) concentration measured by using Jaffé method [23], a calibrator traceable to the international standardization with isotope dilution mass spectrometry (IDMS) [24]. Plasma cystatin C (mg/L) was analyzed with a particle-enhanced immunonephelometric assay (N Latex Cystatin; Dade Behring, Deerfield, IL, USA). The values for cystatin C were analyzed before the introduction of the world calibrator in 2010 and thus not standardized [25]. The standardization of cystatin C values was performed as described elsewhere [26].

### Estimation of kidney function

Estimated glomerular filtration rate (eGFR) was calculated by using seven different equations: Chronic Kidney Disease Epidemiology Collaboration (CKD-EPI) 2012 creatinine and cystatin C equations [24] (CKD-EPI_CR_, CKD-EPI_CYS_, CKD-EPI_CR-CYS_))_,_ cystatin C eGFR equation based on Caucasian, Asian, pediatric, and adult cohorts (CAPA) [25], the Lund-Malmö revised creatinine based eGFR equation (LMrev) [27], modified Full Age Spectrum creatinine-based equation (EKFC_CR_) [28] and combined CAPA-LMrev equation.

Urinary albumin-to-creatinine ratio (mg/mmol) (UACR) was measured in morning urine samples as described previously [29].

### Statement of ethics

Each investigator committed to comply with legislation and to conduct the study in line with regulations, in accordance with the 1975 Declaration of Helsinki and its later amendments. The following ethical permissions apply for the cohort: MDCS (LU 51/90, 532/2006). All participants signed the informed consent.

### Statistical analysis

Statistical analysis was conducted by using Rcrmdr version R 3.6.2 GUI 1.70 El Capitan build macOS. A p-value less than 0.05 was considered significant.

Continuous variables are expressed as mean and standard deviations (SD), discrete variables as medians with interquartile ratio (IQR) and categorical variables as numbers and percentages in parentheses. F-test was applied to test the equality of two populations for normally distributed continuous data, and only after this a Student’s t-test was performed. Two groups of not normally distributed data were compared by using nonparametric two-sample Wilcoxon test. When applicable, a Chi-square test was conducted. Spearman correlation test was carried out to define the correlations among variables if at least one of variables was not normally distributed. Adjusted *P* values (Holm-Bonferroni method) were used in order to counteract the problem of multiple comparisons.

Since the difference between men and women in baseline clinical parameters was significant the interaction analysis was performed. We checked if interaction between sex, fat mass index and mean arterial pressure was associated with different equations for eGFR. For this fat mass index z-score and the centralized values for mean arterial pressure were calculated and then put in the multiple linear regression.

To find out which factors that reflect body composition are associated with estimated kidney function following adjustments for age, sex and mean arterial pressure the multivariable linear regression was accomplished.

## Results

### Participants

**Fig 1:**
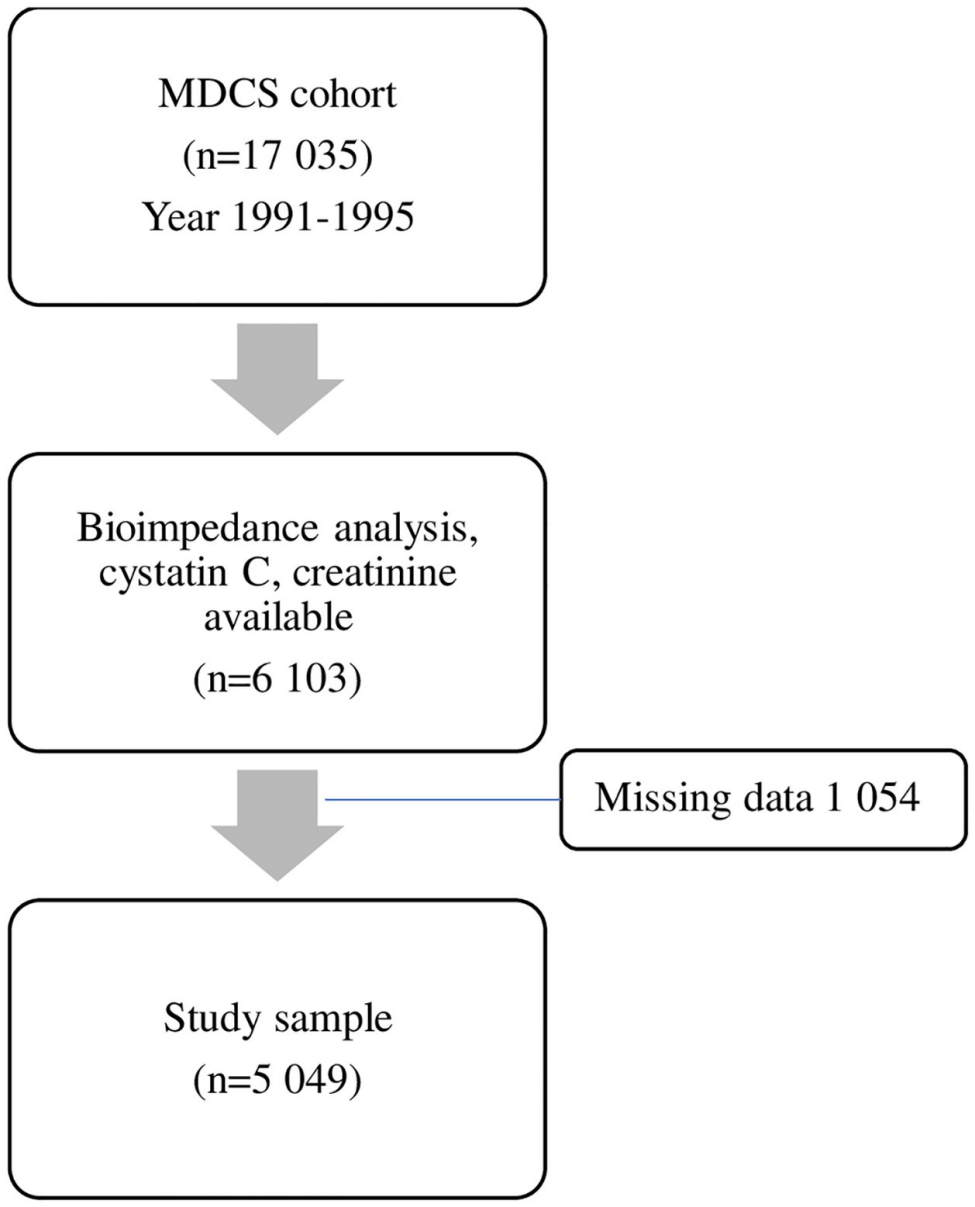
Selection of subjects for a study. MDCS, Malmo Diet Cancer study.

The flow chart of this study is presented in Fig 1.

### General characteristics of the study population

The general demographic characteristics and comparison of clinical characteristics between men and women are presented in Tables 1 and 2, respectively.

**Table 1:** Demographic characteristics of study subjects.

Variable	Total (n = 5049)	Women (n = 2990)	Men (n = 2059)
Age, at baseline, years	57 (6)	57 (6)	57 (6)
Age, at follow-up, years	73 (6)	73 (6)	73 (6)
Diabetes, yes	668 (13.2)	412 (13.8)	256 (12.4)
Hypertension, yes	1889 (22.3)	1028 (34.3)	861 (41.8)
Obesity, yes	651 (12.9)	401 (13.4)	250 (12.1)
Lipid-lowering treatment, at follow-up, yes	120 (2.9)	55 (1.8)	65 (3.1)
AntiHT treatment^a^, at follow-up, yes	831 (36.7)^a^	466 (56.1)^a^	365 (43.9)^a^
Smoking, yes	1085 (21.5)	628 (21.0)	457 (22.1)

Data expressed as number (%).

^a^ data available in total of 2265 subjects: 1513 women and 752 men.

Abbreviations: AntiHT, antihypertensive treatment.

Smoking determined as current active smoking; obesity as BMI ≥30 kg/m^2^.

**Table 2:** Clinical characteristics and comparisons between men and women at baseline.

Variable	Total (n = 5049)	Women (n = 2990)	Men (n = 2059)	*P*
BMI, kg/m^2^	25.7 (3.9)	25.4 (4.2)	26.2 (3.5)	<0.001
FM, kg	20.0 (6.8)	21.7 (6.7)	17.5 (6.2)	<0.001
LM, kg	52.9 (10.9)	45.8 (5.6)	63.4 (8.1)	<0.001
Body fat %	27 (7)	31 (5)	21 (5)	<0.001
FMI, kg/m^2^	7.1 (2.6)	8.1 (2.5)	5.6 (2.0)	<0.001
z-score FMI_Larsson_^a^	-0.53 (0.85)	-0.47 (0.79)	-0.62 (0.91)	<0.001
z-score FMI_Lee_^b^	-1.45 (2.51)	-1.32 (2.42)	-1.64 (2.63)	<0.001
TBW, L	37.9 (7.4)	32.7 (3.1)	45.5 (4.8)	<0.001
WC, cm	84 (13)	77 (10)	93 (10)	<0.001
WHR	0.85 (0.09)	0.79 (0.05)	0.94 (0.06)	<0.001
SBP, mmHg	141 (19)	141 (19)	143 (19)	<0.001
DBP, mmHg	87 (9)	86 (9)	89 (10)	<0.001
MAP, mmHg	105 (12)	104 (11)	107 (12)	<0.001
Cystatin C, mg/L	1.17 (0.29)	1.18 (0.28)	1.17 (0.29)	0.005
Creatinine, µmol/L	85 (16)	79 (13)	93 (18)	<0.001
CAPA, mL/min/1.73m^2^	64 (15)	64 (15)	65 (15)	0.005
CKD-EPI_CYS,_ mL/min/1.73m^2^	69 (16)	63 (17)	77 (8)	<0.001
LMrev, mL/min/1.73m^2^	77 (13)	82 (12)	71 (12)	<0.001
CKD-EPI_CR,_ mL/min/1.73m^2^	76 (14)	74 (13)	76 (9)	<0.001
EKFC_CR_, mL/min/1.73m^2^	69 (14)	80 (14)	79 (14)	<0.001
CKD-EPI_CR-CYS,_ mL/min/1.73m^2^	79 (14)	67 (14)	73 (14)	<0.001
CAPA-LMrev, mL/min/1.73m^2^	71 (12)	73 (12)	68 (12)	<0.001
UACR, mg/mmol	2.36 (11.04)	1.54 (6.04)	3.45 (15.28)	0.830

Data expressed as Mean (± SD) or Median with interquartile ratio (IQR).

^a^ based on DEXA acquired fat mass.

^b^ based on bioimpedance acquired fat mass.

Abbreviations: BMI, body mass index; F: fasting; FM, fat mass; LM, lean mass; FMI, fat mass index; TBW, total body water; WC, waist circumference; WHR, waist-to-hip ratio; SBP, systolic blood pressure; DBP, diastolic blood pressure; MAP, mean arterial pressure; eGFR, estimated glomerular filtration rate; CAPA, cystatin C eGFR equation based on Caucasian, Asian, pediatric, and adult cohorts; LMrev, the Lund-Malmö revised creatinine based eGFR equation; CKD-EPI_CYS,_ the Chronic Kidney Disease Epidemiology Collaboration cystatin C equation; CKD-EPI_CR,_ the Chronic Kidney Disease Epidemiology Collaboration creatinine equation; CKD-EPI_CR-CYS,_ the Chronic Kidney Disease Epidemiology Collaboration combined creatinine and cystatin C equation; EKFC_CR_, a Modified Full Age Spectrum creatinine-based equation; CAPA-LMrev, average eGFR calculated from CAPA and LMrev; UACR, urinary albumin to creatinine ratio.

### Body mass index and kidney function

Overweight (n = 2000) and/or obesity (n = 651) were associated with decreased eGFR, irrespective of sex. Obese subjects vs. overweight vs. normal BMI had *lower* CAPA (66 vs. 63 vs. 60 mL/min/1.73m^2^ respectively, p<0.001). As for LMrev equation, only overweight subjects had *lower* LMrev as compared either to obese subjects or to those with normal BMI (76 vs.78 mL/min/1.73m^2^, p = 0.003 and 76 vs 79 mL/min/1.73m^2^, p<0.001 respectively). The latter observations were not present in CKD-EPI_CR_ equation. CKD-EPI_CYS_ but not CAPA equation revealed sex- and BMI interactions, showing significantly *lower* eGFR among overweight/obese women (61 and 57 mL/min/1.73m^2^, p<0.001) compared to men (77 and 76 mL/min/1.73m^2^, p<0.001). These associations–except for the LM rev, CKD-EPI_CR_ and EKFC_CR_ equation—between BMI and kidney function were confirmed by performing linear regression analysis with different eGFR equations as dependent variables following adjustments for age, sex and mean arterial pressure (Table 3). BMI as a continuous variable was associated with eGFR equations that take cystatin C concentration into account. The addition of diabetes to linear regression models did not influence the results due to its low prevalence rate (13.2%).

**Table 3:** Association between body mass index and eGFR equations.

	CAPA			CKD-EPI_CYS_			LMrev			CKD-EPI_CR_		
	**ß**	**SE**	***P***	**ß**	**SE**	***P***	**ß**	**SE**	***P***	**ß**	**SE**	***P***
Age, y	-0.838	0.035	<0.001	-0.893	0.032	<0.001	-0.742	0.027	<0.001	-0.829	0.031	<0.001
Sex, male	1.228	0.415	0.003	14.925	0.382	<0.001	-11.173	0.321	<0.001	6.448	0.367	<0.001
MAP, mmHg	-0.019	0.018	0.298	-0.009	0.017	0.588	-0.010	0.014	0.475	-0.007	0.016	0.666
Overweight, yes (n = 2000)	-2.181	0.441	<0.001	-1.956	0.406	<0.001	-0.409	0.341	0.231	-0.451	0.391	0.248
Obesity, yes (n = 651)	-4.942	0.645	<0.001	-4.667	0.594	<0.001	0.697	0.499	0.162	0.779	0.571	0.172
Age, y	-0.841	0.035	<0.001	-0.896	0.032	<0.001	-0.743	0.027	<0.001	-0.831	0.031	<0.001
Sex. male	1.308	0.411	0.001	15.012	0.379	<0.001	11.259	0.319	<0.001	6.355	0.365	<0.001
MAP, mmHg	-0.013	0.018	0.494	-0.002	0.017	0.862	-0.010	0.014	0.490	-0.007	0.016	0.689
BMI, kg/m^2^	-0.464	0.053	<0.001	-0.043	0.049	<0.001	0.023	0.041	0.566	0.024	0.047	0.604

	EKFC_CR_			CKD-EPI_CR-CYS_			CAPA-LMrev		
	**ß**	**SE**	***P***	**ß**	**SE**	***P***	**ß**	**SE**	***P***
Age, y	-1.068	0.030	<0.001	-0.913	0.031	<0.001	-0.790	0.027	<0.001
Sex, male	-0.950	0.355	0.008	7.071	0.375	<0.001	-4.973	0.324	<0.001
MAP, mmHg	-0.012	0.016	0.431	-0.015	0.017	0.365	-0.015	0.014	0.309
Overweight, yes (n = 2000)	-0.665	0.378	0.079	-1.595	0.399	<0.001	-1.295	0.345	<0.001
Obesity, yes (n = 651)	0.223	0.552	0.686	-2.842	0.584	<0.001	-2.123	0.501	<0.001
Age, y	-1.070	0.030	<0.001	-0.916	0.031	<0.001	-0.792	0.027	<0.001
Sex, male	-1.040	0.353	0.003	7.079	0.373	<0.001	-4.976	0.322	<0.001
MAP, mmHg	-0.013	0.016	0.421	-0.011	0.017	0.510	-0.011	0.014	0.437
BMI, kg/m^2^	-0.008	0.045	0.857	-0.284	0.478	<0.001	-0.220	0.041	<0.001

Multiple linear regression analysis, eGFR equations (dependent variable) following adjustments for age, sex and mean arterial pressure.

Where applicable normal body mass index group used as a reference value.

Abbreviations: BMI, body mass index; eGFR, estimated glomerular filtration rate; CAPA, cystatin C eGFR equation based on Caucasian, Asian, pediatric, and adult cohorts; LMrev, the Lund-Malmö revised creatinine based eGFR equation; CKD-EPI_CYS,_ the Chronic Kidney Disease Epidemiology Collaboration cystatin C equation; CKD-EPI_CR_, the Chronic Kidney Disease Epidemiology Collaboration creatinine equation; CKD-EPI_CR-CYS,_ the Chronic Kidney Disease Epidemiology Collaboration combined creatinine and cystatin C equation; EKFC_CR_, a Modified Full Age Spectrum creatinine-based equation; CAPA-LMrev, average eGFR calculated from CAPA and LMrev.

### Fat mass and kidney function

Body impedance acquired measurement and anthropometric parameter correlations with different eGFR equations, as well as with cystatin C and creatinine concentrations, in men and women are listed as S1 Table. The strongest significant correlations were as follows: fat mass, FMI, z-score FMI_Lee_ and WC negatively correlated with both cystatin C equations, and positively with cystatin C concentration in both men and women, whereas combined creatinine and cystatin C equations were negatively correlated to fat mass, FMI and WC only. Lean mass was negatively associated with CKD-EPI_CR_ and positively with plasma creatinine concentration in men.

The interaction analysis could not confirm neither interrelationship between sex and FMI, nor between sex, MAP and FMI z-scores that is associated with eGFR equations (S2 Table). Therefore, the differences between sexes were addressed with caution.

In multivariable linear regression models (Table 4) a 1 kg/m^2^ increase in FMI was associated with a decrease in predicted CAPA by 0.77 mL/min/1.73m^2^, a decrease in predicted CKD-EPI_CYS_ by 0.73 mL/min/1.73m^2^, as well as in CDK-EPI_CR-CYS_ by 0.36 mL/min/1.73m^2^ and in CAPA-LMrev by 0.26 mL/min/1.73m^2^. Furthermore, increase in FMI z-score_Larsson_ or FMI z-score_Lee_ was related to a decrease in predicted CAPA by 2.02 and 0.69 mL/min/1.73m^2^, respectively, a decrease in predicted CKD-EPI_CYS_ by 1.81 and 0.62 mL/min/1.73m^2^, as well as in predicted CDK-EPI_CR-CYS_ by 0.90 and 0.32 mL/min/1.73m^2^ and in CAPA-LMrev by 0.65 and 0.23 mL/min/1.73m^2^, respectively. The association with eGFR based on creatinine–LM rev, EKFC_CR_ and CKD-EPI_CR_−was much weaker though statistically significant; showing that an increase in FMI and its z-scores was related to an increase in kidney function derived from eGFR_CR_ equations. The addition of diabetes mellitus to the models did not affect the results provided (not listed in a Table 4).

**Table 4:** Association between fat mass index z-scores and eGFR equations.

	CAPA			CKD-EPI_CYS_			LMrev			CKD-EPI_CR_		
	**ß**	**SE**	***P***	**ß**	**SE**	***P***	**ß**	**SE**	***P***	**ß**	**SE**	***P***
Age, y	-0.831	0.035	<0.001	-0.886	0.032	<0.001	-0.749	0.027	<0.001	-0.84	0.03	<0.001
Sex, male	-0.972	0.475	0.041	12.846	0.437	<0.001	-10.597	0.367	<0.001	7.12	0.42	<0.001
MAP, mmHg	-0.014	0.018	0.449	-0.004	0.017	0.828	-0.020	0.014	0.166	-0.02	0.02	0.26
FMI, kg/m^2^	-0.768	0.089	<0.001	-0.731	0.082	<0.001	0.243	0.069	<0.001	0.28	0.08	<0.001
Age, y	-0.899	0.035	<0.001	-0.948	0.032	<0.001	-0.726	0.027	<0.001	-0.811	0.031	<0.001
Sex, male	0.652	0.414	0.116	14.416	0.382	<0.001	-11.195	0.320	<0.001	6.543	0.366	<0.001
MAP, mmHg	-0.015	0.018	0.408	-0.007	0.017	0.678	-0.021	0.014	0.147	-0.019	0.016	0.236
z-score FMI_Larsson_	-2.023	0.245	<0.001	-1.806	0.226	<0.001	0.716	0.189	0.001	0.834	0.217	0.0001
Age, y	-0.868	0.035	<0.001	-0.920	0.032	<0.001	-0.737	0.027	<0.001	-0.824	0.031	<0.001
Sex, male	0.745	0.412	<0.001	14.500	0.380	<0.001	-11.138	0.319	<0.001	6.491	0.365	<0.001
MAP, mmHg	-0.014	0.018	0.448	-0.006	0.017	0.721	-0.020	0.014	0.161	-0.018	0.016	0.263
z-score FMI_Lee_	-0.694	0.082	<0.001	-0.616	0.076	<0.001	0.225	0.064	<0.001	0.256	0.073	<0.001

	EKFC_CR_			CKD-EPI_CR-CYS_			CAPA-LMrev		
	**ß**	**SE**	***P***	**ß**	**SE**	***P***	**ß**	**SE**	***P***
Age, y	-1.076	0.030	<0.001	-0.913	0.031	<0.001	-0.790	0.027	<0.001
Sex, male	-0.500	0.407	0.218	5.984	0.430	<0.001	-5.784	0.372	<0.001
MAP, mmHg	-0.023	0.016	0.141	-0.017	0.017	0.302	-0.017	0.014	0.244
FMI, kg/m^2^	0.203	0.076	0.008	-0.358	0.081	<0.001	-0.262	0.070	<0.001
Age, y	-1.056	0.030	<0.001	-0.944	0.032	<0.001	-0.812	0.027	<0.001
Sex, male	-0.909	0.355	0.010	6.750	0.376	<0.001	-5.222	0.325	<0.001
MAP, mmHg	-0.025	0.016	0.118	-0.019	0.017	0.266	-0.018	0.014	0.214
z-score FMI_Larsson_	0.635	0.210	0.002	-0.901	0.222	<0.001	-0.654	0.192	0.001
Age, y	-1.066	0.030	<0.001	-0.930	0.031	<0.001	-0.803	0.027	<0.001
Sex, male	-0.949	0.353	0.007	6.783	0.374	<0.001	-5.200	0.323	<0.001
MAP, mmHg	-0.024	0.016	0.130	-0.017	0.017	0.304	-0.017	0.014	0.240
z-score FMI_Lee_	0.196	0.070	0.005	-0.325	0.075	<0.001	-0.234	0.064	<0.001

Multiple linear regression analysis, eGFR equations (dependent variable) following adjustments for age, sex and mean arterial pressure.

Abbreviations: FMI, fat mass index; eGFR, estimated glomerular filtration rate; CAPA, cystatin C eGFR equation based on Caucasian, Asian, pediatric, and adult cohorts; LMrev, the Lund-Malmö revised creatinine based eGFR equation; CKD-EPI_CYS_, the Chronic Kidney Disease Epidemiology Collaboration cystatin C equation; CKD-EPI_CR_, the Chronic Kidney Disease Epidemiology Collaboration creatinine equation; CKD-EPI_CR-CYS,_ the Chronic Kidney Disease Epidemiology Collaboration combined creatinine and cystatin C equation; EKFC_CR_, a Modified Full Age Spectrum creatinine-based equation, CAPA-LMrev, average eGFR calculated from CAPA and LMrev.

## Discussion and conclusions

The introduction of cystatin C, as a marker of kidney function, has simplified early detection of CKD. Though, the use of eGFR in obese population is less well studied. Here we show that fat mass index and its z-scores are strongly associated with *lower* cystatin C based eGFR equations possibly indicating either early kidney damage with altered glomerular permeability or cystatin C production by adipose tissue. The association between fat mass index and creatinine-based eGFR formulas was much weaker.

The interaction between adipose and renal tissue, the so called adipo-renal axis, is critical for kidney health [4]. This bi-directional crosstalk complicates the diagnosis of kidney lesion in obesity since it raises doubts as to whether altered immunologic and endocrine function of adipose tissue has led to kidney damage, or the underlying kidney disease has exacerbated obesity. The identifications of body composition and fat distribution has become a game changer in adiposity evaluation in relation to CKD, and helps to address the “obesity paradox” based on BMI calculation in end stage renal disease (ESRD) [3]. The “obesity paradox” defines beneficial effect of increasing BMI on survival in end-stage kidney disease [30].

In this observational cohort study, we show that both *increased* BMI and fat mass index were associated with significantly *lower* cystatin C derived eGFR, irrespective of sex. Moreover, *decreased* cystatin C based kidney function and *increased* cystatin C concentration *per se* correlated with *increased* fat mass and abdominal obesity. Obesity induced glomerular damage is one of possible explanations [31], since 2651 from 5049 subjects in our study were either overweight or obese. Indeed, kidney biopsies in obese subjects [31] report glomerular basement membrane thickening in addition to glomerulosclerosis. These histological changes are like those observed in Shrunken Pore Syndrome (SPS) [32]. Increased thickness of glomerular basement membrane alters glomerular permeability and might cause reduced clearance of middle-sized weight proteins (10–30 kDa) [33], including cystatin C and atherosclerosis-promoting proteins [34]. This possible link between increased cystatin C and atherosclerosis needs further exploration.

On the other side, cystatin C expression in adipose tissue, confirmed by both animal [35] and human studies [13], might also affect a possible underestimation of kidney function in studied populations. Human adipose tissue biopsy data showed 3-fold higher cystatin C expression in adipose stromal cells in obese subjects as compared to non-obese [13, 14]. Though these findings need further elucidation.

The mGFR (based on iohexol or inulin clearance) might underestimate kidney function if adjusted to body surface area using real body weight instead of ideal body weight [36]. CKD-EPI_CR_ as compared to mGFR has been reported to be valid in obesity, especially for CKD stage 3 to 5 [36]. However, both MDRD (The Modification of Diet in Renal Disease) and CKD-EPI_CR_ underestimates kidney function if mGFR is lower than 30 ml/min/1,73m^2^ [37]. Our finding that increased FMI is related to an increase in predicted kidney function derived from creatinine-based eGFR equations might be explained by the previous observations: (i) increased creatinine excretion and generation in obesity [38]; (ii) obesity related renal hyperfiltration–an early sign of chronic kidney damage [39].

In terms of drug prescription, the underestimation of kidney function in obesity prevents overdosing, though, underdosing can result in therapy failure [40]. Interestingly, CKD-EPI_CR-CYS_ has better performance in evaluating kidney function as compared to either CKD-EPI_CR_ or CKD-EPI_CYS_ in patients undergoing bariatric surgery [41].

The strength of this study is that we used a population-based cohort and that it includes different eGFR equations, currently widely used in clinical practice, with body composition measurements. We calculated FMI z-scores based on reference values for bioimpedance and for DEXA and therefore limiting the chance of imprecise result interpretation.

Limitations of the study is the use of bioimpedance performed by single-frequency equipment in 1991–1996. Multifrequency bioimpedance measurements or DEXA are more accurate techniques for evaluating the proposed three-compartment body composition model [42]. We did not have data on measured GFR (iohexol clearance) to clarify whether the cystatin C and creatinine based eGFR was under-/overestimated in our population [43], whilst this method is not suitable for large cohort studies.

In summary, here we show that cystatin C based eGFR equations could better reveal relationship between fat mass index and its z-scores and kidney function. The association between fat mass index and combined cystatin C and creatinine based eGFR equations and creatinine-based eGFR formulas was much weaker.

### Future perspectives

The association between cystatin C and fat mass may be due to several reasons. Obesity induced kidney damage is one. Increased cystatin C concentration against the background of different permeability of middle-sized molecules in kidney is the second and can be coupled to cardiorenal diseases. Finally, further human studies are warranted to elucidate whether cystatin C originates from adipose tissue.

## Supporting information

S1 Table Estimated glomerular filtration rate correlations with body composition and anthropometric measurements in men and women. (DOCX)

S2 Table Multiple linear regression, interaction analysis. eGFR equations (dependent variable) following adjustments for age. sex and centralized mean arterial pressure.
(DOCX)
