# Supplementary material for: Increased fat mass index is associated with decreased glomerular filtration rate estimated from cystatin C. Data from Malmö Diet and Cancer cohort
Source: PLoS One. 2022 Jul 21;17(7):e0271638. doi: 10.1371/journal.pone.0271638 (PMC9302820; doi:10.1371/journal.pone.0271638)
Supplement: S1 Table — (DOCX) [file pone.0271638.s001.docx]

**S1 Table. Estimated glomerular filtration rate correlations with body composition and anthropometric measurements in men and women**

| **MEN** | **CAPA** | | **CKD-EPI_CYS_** | | **LMrev** | | **CKD-EPI_CR_** | | **FAS_CR_** | | **CKD-EPI_CR-CYS_** | | **CAPA/LMrev** | | **Cystatin C** | | **Creatinine** | |
| --- | --- | --- | --- | --- | --- | --- | --- | --- | --- | --- | --- | --- | --- | --- | --- | --- | --- | --- |
|  | r | *P* | r | *P* | r | *P* | r | *P* | r | *P* | r | *P* | r | *P* | r | *P* | r | *P* |
| FM | -0.15 | <0.001 | -0.15 | <0.001 | -0.34 | 1.000 | -0.03 | <0.001 | -0.03 | 0.147 | -0.11 | <0.001 | -0.10 | <0.001 | 0.15 | <0.001 | 0.02 | 1.000 |
| LM | -0.06 | 0.126 | -0.04 | 1.000 | -0.09 | 0.006 | -0.10 | 0.005 | -0.08 | 0.001 | -0.08 | 0.0003 | -0.08 | 0.0002 | 0.07 | 0.069 | 0.12 | <0.001 |
| FMI | -0.15 | <0.001 | -0.15 | <0.001 | -0.03 | 1.000 | -0.03 | 1.000 | -0.03 | 0.133 | -0.11 | <0.001 | -0.10 | <0.001 | 0.15 | <0.001 | 0.01 | 1.000 |
| z-score FMI_Larsson_ | -0.06 | 0.163 | -0.02 | 1.000 | 0.07 | 0.043 | 0.07 | 0.043 | 0.09 | <0.001 | -0.003 | 0.884 | 0.003 | 0.889 | 0.07 | 0.040 | -0.03 | 1.000 |
| z-score FMI_Lee_ | -0.11 | <0.001 | -0.10 | <0.001 | 0.01 | 1.000 | 0.01 | 1.000 | 0.02 | 0.473 | -0.07 | 0.002 | -0.06 | 0.006 | 0.12 | <0.001 | -0.01 | 1.000 |
| WC | -0.14 | <0.001 | -0.14 | <0.001 | -0.05 | 0.287 | -0.06 | 0.273 | -0.06 | 0.007 | -0.12 | <0.001 | -0.11 | <0.001 | 0.14 | <0.001 | 0.03 | 1.000 |
| WHR | -0.06 | 0.097 | -0.05 | 0.187 | 0.04 | 1.000 | 0.04 | 1.000 | 0.04 | 0.080 | -0.03 | 0.243 | -0.02 | 0.407 | 0.07 | 0.076 | -0.03 | 0.832 |

| **WOMEN** | **CAPA** | | **CKD-EPI_CYS_** | | **LMrev** | | **CKD-EPI_CR_** | | **FAS_CR_** | | **CKD-EPI_CR-CYS_** | | **CAPA/LMrev** | | **Cystatin C** | | **Creatinine** | |
| --- | --- | --- | --- | --- | --- | --- | --- | --- | --- | --- | --- | --- | --- | --- | --- | --- | --- | --- |
|  | r | *P* | r | *P* | r | *P* | r | *P* | r | *P* | r | *P* | r | *P* | r | *P* | r | *P* |
| FM | -0.11 | <0.001 | -0.19 | <0.001 | -0.02 | 1.000 | -0.02 | 1.000 | -0.07 | 0.0003 | -0.13 | <0.001 | -0.13 | <0.001 | 0.19 | <0.001 | -0.01 | 1.000 |
| LM | -0.10 | <0.001 | -0.09 | <0.001 | -0.02 | 1.000 | -0.02 | 1.000 | 0.01 | 0.600 | -0.07 | <0.001 | -0.07 | 0.0003 | 0.10 | <0.001 | 0.04 | 0.289 |
| FMI | -0.20 | <0.001 | -0.20 | <0.001 | -0.03 | 1.000 | -0.02 | 1.000 | -0.09 | <0.001 | -0.14 | <0.001 | -0.14 | <0.001 | 0.20 | <0.001 | -0.02 | 1.000 |
| z-score FMI_Larsson_ | -0.11 | <0.001 | -0.11 | <0.001 | 0.07 | 0.004 | 0.06 | 0.004 | 0.07 | 0.0002 | -0.04 | 0.020 | -0.04 | 0.058 | 0.12 | <0.001 | -0.06 | 0.018 |
| z-score FMI_Lee_ | -0.15 | <0.001 | -0.15 | <0.001 | 0.02 | 1.000 | 0.03 | 1.000 | -0.01 | 0.995 | -0.09 | <0.001 | -0.08 | <0.001 | 0.15 | <0.001 | -0.04 | 0.344 |
| WC | -0.18 | <0.001 | -0.18 | <0.001 | -0.03 | 1.000 | -0.02 | 1.000 | 0.07 | <0.001 | -0.13 | <0.001 | -0.12 | <0.001 | 0.17 | <0.001 | -0.01 | 1.000 |
| WHR | -0.07 | 0.009 | -0.06 | 0.010 | 0.04 | 0.497 | 0.04 | 0.453 | 0.03 | 0.164 | -0.03 | 0.134 | -0.02 | 0.198 | 0.07 | 0.0081 | -0.04 | 0.344 |

Adjusted *P* values (Holm-Bonferoni method) was used to counteract the problem of multiple comparisons.

Abbreviations: FM, fat mass; LM, lean mass; FMI, fat mass index; WC, waist circumference; WHR, waist-to-hip ratio; CAPA, cystatin C eGFR equation based on Caucasian, Asian, pediatric, and adult cohorts; LMrev, the Lund-Malmö revised creatinine based eGFR equation; CKD-EPI_CYS,_ the Chronic Kidney Disease Epidemiology Collaboration cystatin C equation; CKD-EPI_CR_, the Chronic Kidney Disease Epidemiology Collaboration creatinine equation; CKD-EPI_CR-CYS,_ the Chronic Kidney Disease Epidemiology Collaboration combined creatinine and cystatin C equation;FAS, Full Age Spectrum creatinine-based equation ; z-score FMI_Larsson_, z score for fat mass index calculated by using references as reported by Larsson et al.; z-score FMI_Lee_, z score for fat mass index calculated by using references as reported by Lee et al
