# Supplementary material for: Increased fat mass index is associated with decreased glomerular filtration rate estimated from cystatin C. Data from Malmö Diet and Cancer cohort
Source: PLoS One. 2022 Jul 21;17(7):e0271638. doi: 10.1371/journal.pone.0271638 (PMC9302820; doi:10.1371/journal.pone.0271638)
Supplement: S2 Table — eGFR equations (dependent variable) following adjustments for age. sex and centralized mean arterial pressure. (DOCX) [file pone.0271638.s002.docx]

**S2 Table. Multiple linear regression, interaction analysis. eGFR equations (dependent variable) following adjustments for age. sex and centralized mean arterial pressure.**

**CAPA**

|  | **ß** | **SE** | ***P*** |
| --- | --- | --- | --- |
| (Intercept) | 113.24 | 3.21 | <0.001 |
| Age (years) | -0.90 | 0.04 | <0.001 |
| Sex (men) | 1.08 | 4.65 | 0.82 |
| MAP_centralized | 1.04 | 2.89 | 0.72 |
| FMI z-score_Larsson_ | -4.48 | 3.08 | 0.15 |
| FMI z-score_Larsson_ *MAP_centralized | 2.18 | 3.05 | 0.48 |
| Sex (men)*MAP_centralized | -0.45 | 4.47 | 0.92 |
| Sex (men)* FMI z-score_Larsson_ | -4.48 | 4.34 | 0.30 |
| Sex (men)* FMI z-score_Larsson_ *MAP_centralized | 4.67 | 4.19 | 0.26 |

|  | **ß** | **SE** | ***P*** |
| --- | --- | --- | --- |
| (Intercept) | 116.25 | 3.28 | <0.001 |
| Age (years) | -0.86 | 0.03 | <0.001 |
| Sex (men) | 6.09 | 4.51 | 0.18 |
| MAP_centralized | -1.79 | 3.02 | 0.55 |
| FMI z-score_Lee_ | -5.61 | 3.29 | 0.09 |
| FMI z-score_Larsson_ *MAP_centralized | 3.10 | 3.24 | 0.34 |
| Sex (men)*MAP_centralized | -5.64 | 4.52 | 0.21 |
| Sex (men)* FMI z-score_Lee_ | -0.82 | 5.33 | 0.88 |
| Sex (men)* FMI z-score_Lee_ *MAP_centralized | 1.55 | 5.13 | 0.76 |

**CKD-EPI_CYS_**

|  | **ß** | **SE** | ***P*** |
| --- | --- | --- | --- |
| (Intercept) | 116.39 | 2.96 | <0.001 |
| Age (years) | -0.94 | 0.03 | <0.001 |
| Sex (men) | 11.13 | 4.28 | 0.01 |
| MAP_centralized | -0.89 | 2.66 | 0.74 |
| FMI z-score_Larsson_ | -4.87 | 2.84 | 0.09 |
| FMI z-score_Larsson_ *MAP_centralized | 2.50 | 2.81 | 0.37 |
| Sex (men)*MAP_centralized | 3.79 | 4.11 | 0.36 |
| Sex (men)* FMI z-score_Larsson_ | 0.50 | 3.99 | 0.90 |
| Sex (men)* FMI z-score_Larsson_ *MAP_centralized | 0.52 | 3.85 | 0.89 |

|  | **ß** | **SE** | ***P*** |
| --- | --- | --- | --- |
| (Intercept) | 120.09 | 3.02 | <0.001 |
| Age (years) | -0.91 | 0.03 | <0.001 |
| Sex (men) | 10.62 | 4.14 | 0.01 |
| MAP_centralized | -3.94 | 2.78 | 0.16 |
| FMI z-score_Lee_ | -6.39 | 3.02 | 0.03 |
| FMI z-score_Larsson_ *MAP_centralized | 3.69 | 2.98 | 0.22 |
| Sex (men)*MAP_centralized | 3.10 | 4.15 | 0.45 |
| Sex (men)* FMI z-score_Lee_ | 4.52 | 4.90 | 0.36 |
| Sex (men)* FMI z-score_Lee_ *MAP_centralized | -2.75 | 4.72 | 0.56 |

**LM_REV_**

|  | **ß** | **SE** | ***P*** |
| --- | --- | --- | --- |
| (Intercept) | 125.14 | 2.48 | <0.001 |
| Age (years) | -0.73 | 0.03 | <0.001 |
| Sex (men) | -12.50 | 3.59 | 0.00 |
| MAP_centralized | -1.18 | 2.24 | 0.60 |
| FMI z-score_Larsson_ | 0.30 | 2.38 | 0.90 |
| FMI z-score_Larsson_ *MAP_centralized | 0.62 | 2.36 | 0.79 |
| Sex (men)*MAP_centralized | 0.93 | 3.45 | 0.79 |
| Sex (men)* FMI z-score_Larsson_ | -4.29 | 3.35 | 0.20 |
| Sex (men)* FMI z-score_Larsson_ *MAP_centralized | 3.61 | 3.24 | 0.26 |

|  | **ß** | **SE** | ***P*** |
| --- | --- | --- | --- |
| (Intercept) | 126.06 | 2.53 | <0.001 |
| Age (years) | -0.74 | 0.03 | <0.001 |
| Sex (men) | -8.36 | 3.48 | 0.02 |
| MAP_centralized | -2.33 | 2.33 | 0.32 |
| FMI z-score_Lee_ | -1.25 | 2.54 | 0.62 |
| FMI z-score_Larsson_ *MAP_centralized | 2.05 | 2.50 | 0.41 |
| Sex (men)*MAP_centralized | -2.70 | 3.49 | 0.44 |
| Sex (men)* FMI z-score_Lee_ | -2.69 | 4.11 | 0.51 |
| Sex (men)* FMI z-score_Lee_ *MAP_centralized | 2.43 | 3.96 | 0.54 |

**CKD-EPI_CR_**

|  | **ß** | **SE** | ***P*** |
| --- | --- | --- | --- |
| (Intercept) | 121.37 | 2.84 | <0.001 |
| Age (years) | -0.81 | 0.03 | <0.001 |
| Sex (men) | 5.95 | 4.11 | 0.15 |
| MAP_centralized | -0.73 | 2.56 | 0.78 |
| FMI z-score_Larsson_ | 1.31 | 2.73 | 0.63 |
| FMI z-score_Larsson_ *MAP_centralized | -0.27 | 2.70 | 0.92 |
| Sex (men)*MAP_centralized | 0.07 | 3.95 | 0.99 |
| Sex (men)* FMI z-score_Larsson_ | -5.97 | 3.84 | 0.12 |
| Sex (men)* FMI z-score_Larsson_ *MAP_centralized | 5.26 | 3.70 | 0.16 |

|  | **ß** | **SE** | ***P*** |
| --- | --- | --- | --- |
| (Intercept) | 121.72 | 2.90 | <0.001 |
| Age (years) | -0.83 | 0.03 | <0.001 |
| Sex (men) | 11.92 | 3.98 | 0.00 |
| MAP_centralized | -1.35 | 2.67 | 0.61 |
| FMI z-score_Lee_ | -0.90 | 2.90 | 0.76 |
| FMI z-score_Larsson_ *MAP_centralized | 1.84 | 2.86 | 0.52 |
| Sex (men)*MAP_centralized | -5.33 | 3.99 | 0.18 |
| Sex (men)* FMI z-score_Lee_ | -4.86 | 4.71 | 0.30 |
| Sex (men)* FMI z-score_Lee_ *MAP_centralized | 4.50 | 4.53 | 0.32 |

**FAS_CR_**

|  | **ß** | **SE** | ***P*** |
| --- | --- | --- | --- |
| (Intercept) | 141.84 | 2.75 | <0.001 |
| Age (years) | -1.06 | 0.03 | <0.001 |
| Sex (men) | -2.48 | 3.98 | 0.53 |
| MAP_centralized | -1.45 | 2.48 | 0.56 |
| FMI z-score_Larsson_ | -0.78 | 2.64 | 0.77 |
| FMI z-score_Larsson_ *MAP_centralized | 1.46 | 2.62 | 0.58 |
| Sex (men)*MAP_centralized | 1.26 | 3.82 | 0.74 |
| Sex (men)* FMI z-score_Larsson_ | -3.40 | 3.71 | 0.36 |
| Sex (men)* FMI z-score_Larsson_ *MAP_centralized | 3.02 | 3.59 | 0.40 |

|  | **ß** | **SE** | ***P*** |
| --- | --- | --- | --- |
| (Intercept) | 143.77 | 2.80 | <0.001 |
| Age (years) | -1.07 | 0.03 | <0.001 |
| Sex (men) | 1.46 | 3.85 | 0.71 |
| MAP_centralized | -3.40 | 2.59 | 0.19 |
| FMI z-score_Lee_ | -2.66 | 2.81 | 0.34 |
| FMI z-score_Larsson_ *MAP_centralized | 3.11 | 2.77 | 0.26 |
| Sex (men)*MAP_centralized | -2.57 | 3.86 | 0.51 |
| Sex (men)* FMI z-score_Lee_ | -2.87 | 4.56 | 0.53 |
| Sex (men)* FMI z-score_Lee_ *MAP_centralized | 3.07 | 4.39 | 0.48 |

**CKD-EPI_CR-CYS_**

|  | **ß** | **SE** | ***P*** |
| --- | --- | --- | --- |
| (Intercept) | 119.51 | 2.91 | <0.001 |
| Age (years) | -0.95 | 0.03 | <0.001 |
| Sex (men) | 7.69 | 4.22 | 0.07 |
| MAP_centralized | 0.62 | 2.62 | 0.81 |
| FMI z-score_Larsson_ | -2.04 | 2.79 | 0.47 |
| FMI z-score_Larsson_ *MAP_centralized | 1.03 | 2.77 | 0.71 |
| Sex (men)*MAP_centralized | -1.15 | 4.05 | 0.78 |
| Sex (men)* FMI z-score_Larsson_ | -5.73 | 3.93 | 0.14 |
| Sex (men)* FMI z-score_Larsson_ *MAP_centralized | 5.60 | 3.80 | 0.14 |

|  | **ß** | **SE** | ***P*** |
| --- | --- | --- | --- |
| (Intercept) | 121.40 | 2.97 | <0.001 |
| Age (years) | -0.93 | 0.03 | <0.001 |
| Sex (men) | 13.84 | 4.08 | 0.00 |
| MAP_centralized | -1.27 | 2.74 | 0.64 |
| FMI z-score_Lee_ | -3.75 | 2.98 | 0.21 |
| FMI z-score_Larsson_ *MAP_centralized | 2.59 | 2.93 | 0.38 |
| Sex (men)*MAP_centralized | -7.20 | 4.09 | 0.08 |
| Sex (men)* FMI z-score_Lee_ | -3.31 | 4.83 | 0.49 |
| Sex (men)* FMI z-score_Lee_ *MAP_centralized | 3.61 | 4.65 | 0.44 |

**CAPA-LMrev**

|  | **ß** | **SE** | ***P*** |
| --- | --- | --- | --- |
| (Intercept) | 120.66 | 3.58 | <0.001 |
| Age (years) | -0.81 | 0.03 | <0.001 |
| Sex (men) | 2.58 | 4.89 | 0.60 |
| MAP_centralized | -0.52 | 3.48 | 0.88 |
| FMI z-score_Larsson_ | -0.35 | 3.64 | 0.92 |
| FMI z-score_Larsson_ *MAP_centralized | -7.38 | 4.93 | 0.13 |
| Sex (men)*MAP_centralized | -7.97 | 5.39 | 0.14 |
| Sex (men)* FMI z-score_Larsson_ | -0.58 | 3.66 | 0.87 |
| Sex (men)* FMI z-score_Larsson_ *MAP_centralized | 7.28 | 5.33 | 0.17 |

|  | **ß** | **SE** | ***P*** |
| --- | --- | --- | --- |
| (Intercept) | 121.15 | 2.57 | <0.001 |
| Age (years) | -0.80 | 0.03 | <0.001 |
| Sex (men) | -1.14 | 3.53 | 0.75 |
| MAP_centralized | -2.06 | 2.37 | 0.38 |
| FMI z-score_Lee_ | -3.43 | 2.57 | 0.18 |
| FMI z-score_Larsson_ *MAP_centralized | -4.17 | 3.54 | 0.24 |
| Sex (men)*MAP_centralized | -1.75 | 4.17 | 0.67 |
| Sex (men)* FMI z-score_Lee_ | 2.58 | 2.53 | 0.31 |
| Sex (men)* FMI z-score_Lee_ *MAP_centralized | 1.99 | 4.02 | 0.62 |

CAPA, cystatin C eGFR equation based on Caucasian, Asian, pediatric, and adult cohorts; LMrev, the Lund-Malmö revised creatinine based eGFR equation; CKD-EPI_CYS,_ the Chronic Kidney Disease Epidemiology Collaboration cystatin C equation; CKD-EPI_CR,_ the Chronic Kidney Disease Epidemiology Collaboration creatinine equation; CKD-EPI_CR-CYS,_ the Chronic Kidney Disease Epidemiology Collaboration combined creatinine and cystatin C equation;FAS, Full Age Spectrum creatinine-based equation ; CAPA-LMrev, average eGFR calculated from CAPA and LMrev.
